# Tenuous (in)stability? Mixed policy feedback and its effects on climate policy in Australia and Canada

**DOI:** 10.1007/s11077-026-09609-9

**Published:** 2026-04-29

**Authors:** Jasmin Logg-Scarvell, James Patterson

**Affiliations:** Copernicus Institute of Sustainable Development, Utrecht University, Utrecht, Netherlands

**Keywords:** Policy feedback, Policy stability, Policy conflict, Climate change policy, Carbon pricing

## Abstract

Policy feedback is often viewed as either reinforcing or undermining policy stability. However, it can arise in multiple ways simultaneously, creating mixed policy feedback with complex effects on policy stability. We examine how mixed policy feedback affects policy stability by developing a framework to identify within- and across-level feedbacks, and applying it to two cases of contested climate policy involving domestic carbon markets in Australia (2014–2025) and Canada (2015–2025). In Australia, a nascent baseline-and-credit scheme eventually became entrenched despite initial policy instability, while in Canada, a seemingly stable carbon pricing policy survived early conflict but was later partially reversed. By examining how multiple feedbacks arose and interacted within changing political contexts, we show how mixed policy feedback can affect policy stability, leading to potentially surprising outcomes. This implies a need to go beyond assessing the net effects of mixed policy feedback to consider complex interactions alongside exogenous developments.

**Supplementary Information:**

The online version contains supplementary material available at 10.1007/s11077-026-09609-9.

## Introduction

How is policy stability (i.e., the degree to which policy outputs remain in place) created or undermined through mixed policy feedback? Policy feedback is often viewed as either reinforcing (enhancing policy stability) or undermining (eroding policy stability)^1^ (Campbell, 2012; Pierson, 1993; Mettler & Sorelle, 2018; Oberlander & Weaver, 2015). However, policy feedback can be complex and pull in different directions simultaneously, with indeterminate, unpredictable, and potentially counterintuitive consequences for policy stability (Béland et al., 2019). Such *mixed policy feedback* occurs when multiple elements of a policy (e.g., settings, instruments, ideas) generate feedback that diverges in direction, timeframe, and/or extent (Béland et al., 2022). This requires analysing mixed policy feedback within specific cases to understand its effects on policy stability.

Mixed policy feedback may be especially prevalent in cases of contested policies. Policy feedback is triggered by policy characteristics, but also shaped by power struggles between actors that affect how feedback unfolds over time (Ampe et al., 2021). When a policy is contested, divergent policy positions among actors might strengthen both policy support and opposition, intensifying mixed feedback (Hacker & Pierson, 2019; Stokes, 2020; Weible & Heikkila, 2017). Moreover, policy contestation might shift the focus on different policy elements (e.g., ideas, instruments) or affect the timeframes of feedback (e.g., making it more abrupt or punctuated) (Patashnik, 2019; Patterson & Paterson, 2024). Understanding how mixed policy feedback arises and affects policy stability in contested policy situations is important because of the potential for complex and unexpected feedback combinations.

Therefore, we ask: how does mixed policy feedback affect policy stability in contested policy situations? We develop a framework that disaggregates policy elements to show possible types of feedback within and between these elements, and outline our expectations about feedback direction, strength, and timeframes. While retaining well-established links between feedback direction and policy stability, we suggest that some combinations of mixed feedback might have counterintuitive effects. However, empirical analysis is important because such effects are hard to predict and might also be mediated by exogenous factors (e.g. institutions, culture). We apply our framework to parallel cases of climate policy conflict involving domestic carbon markets in Australia and Canada, drawing on 68 expert interviews. The cases involve broadly similar contexts and degrees of policy conflict, but apparently varying policy stability. We observe complex, mixed policy feedback in both cases, which enhanced policy stability in Australia but eroded it in Canada. Our findings reveal how mixed feedback can temper policy stability, rendering both apparent policy stability and instability tenuous. We also reveal new pathways for counterintuitive effects, with some policy elements generating mixed feedback that concurrently stabilised and destabilised policy at different levels. Overall, we contribute to understanding policy processes by shedding new light on how complex forms of feedback affect policy stability in situations of policy conflict.

The paper proceeds as follows. First, we develop a framework for studying mixed policy feedback within and between policy elements (Sect. 2). We then give key background to our empirical cases and explain our qualitative approach (Sect. 3). We apply our framework to each case to analyse mixed policy feedback and its effect on policy stability (Sect. 4). In the Discussion, we consider the similarities and differences between cases, observing dominant ideas-level feedback being partially countered by weaker feedback at more detailed policy levels, mixed feedback tempering overarching policy stability, and instances where mixed feedback has counterintuitive effects (Sect. 5). We conclude by identifying implications for studying mixed policy feedback within and beyond climate change policymaking, especially regarding stability (Sect. 6). This suggests further research is needed on patterns of mixed feedback across different instruments and the role of strategic actors in shaping policy feedback.

## Mixed policy feedback

**Fig. 1:**
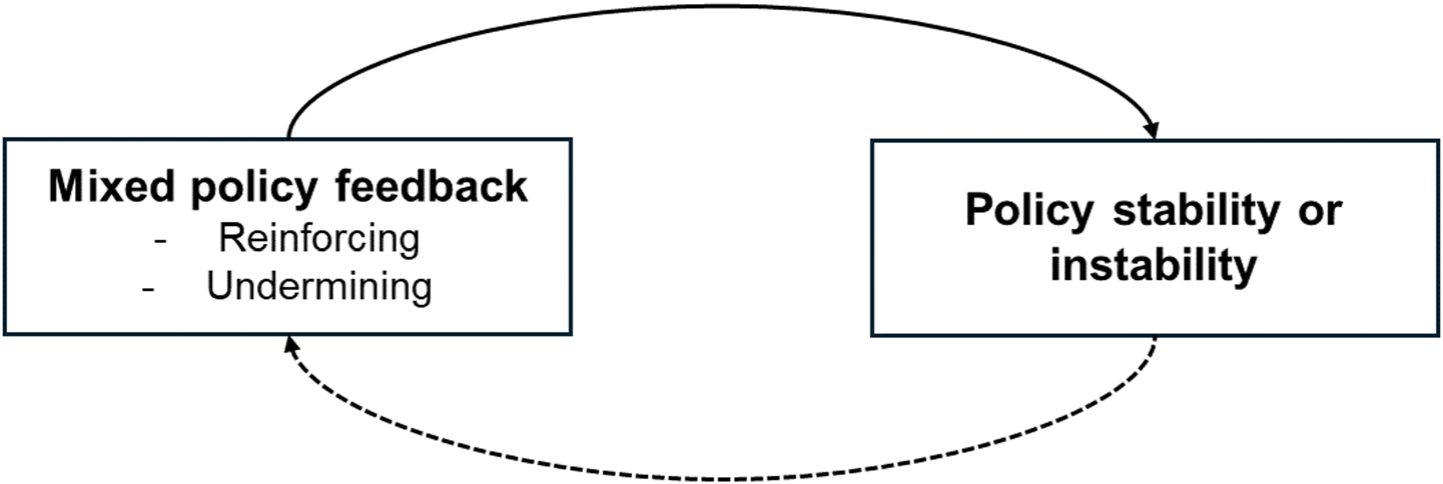
The influence of mixed policy feedback on policy stability (solid arrow indicates our focus, while dashed arrow recognises potential for recursive influence)

Policy feedback scholarship focuses on understanding how policies reshape politics and affect subsequent policymaking (Mettler & Sorelle, 2018). Early work emphasised reinforcing feedback leading to policy stability (Pierson, 1993; Skocpol, 1995). Recent work also examines undermining feedback, where polices produce *in*stability by ‘undoing themselves’ (Jacobs & Weaver, 2015). But what happens when multiple reinforcing and undermining feedbacks co-occur? Reinforcing feedback might dominate due to (perceived or actual) high sunk costs, concentrated benefits, and/or successful delivery (Skogstad, 2020). Alternatively, undermining feedback might dominate due to (perceived or actual) contradictory or unclear goals, concentrated losses, and/or implementation failures (Jacobs & Weaver, 2015). Between these extremes, competing feedbacks may offset each other (Daugbjerg & Kay, 2020). However, it is not just the net effects of reinforcing and/or undermining feedback alone, but also their combinations and interactions that matter for understanding policy stability (Geels & Ayoub, 2025). Over time, policy stability (or instability) may also influence how further feedback is generated (Fig. 1).

**Fig. 2:**
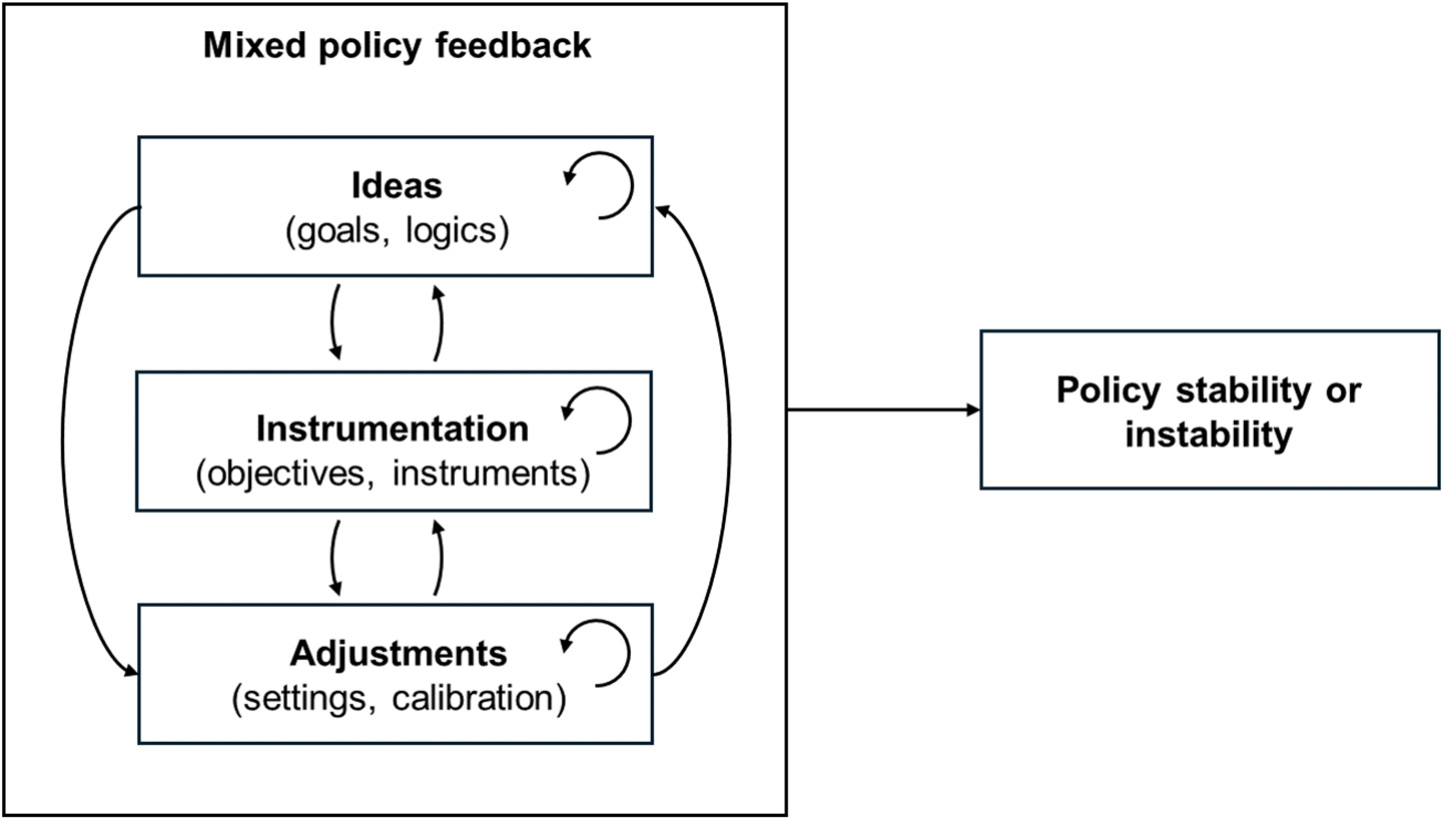
Framework for studying mixed policy feedback, expanding on Fig. 1. Mixed feedbacks are disaggregated within and across different policy elements

Studying mixed policy feedback requires considering ‘policy’ as not a singular object but as comprising multiple interacting elements at different levels of abstraction (Edmondson et al., 2019; Hall, 1993). We therefore use Cashore and Howlett’s (2007) three-level typology: ideas (goals/logics), instrumentation (instruments/objectives), and adjustments (settings/calibrations) to develop our framework for studying mixed policy feedback (Fig. 2). Feedback generated by an element may reinforce or undermine itself or other elements at the same level (within-level feedback). Feedback generated by an element may also reinforce or undermine element/s at another level (across-level feedback). Our approach builds on previous work considering feedback associated with different policy levels/elements (Jordan & Moore, 2023; Moore & Jordan, 2020; Sewerin et al., 2022), yet specifically focuses on the combination of mixed feedback generated within and across policy levels and its impact on policy stability. Although complex, in practice, only a subset of potential feedbacks are likely to manifest empirically in a specific case. Hence, our overarching framework enables analysis to accommodate diverse empirical situations involving different types of feedback within a broad lens, supporting cross-case insights.

We derive initial analytical expectations by reasoning about how mixed feedback could arise and affect policy stability, drawing on key forms of variation in feedback effects proposed by Béland et al. (2022, p.35) concerning strength, duration, and direction. We expect feedback originating from policy ideas (goals, logics) to be the strongest and most persistent, with the largest relative impact on policy stability. Even minor shifts in policy ideas could create significant within- or across-level feedback, such as by placing constraints on policy instruments or calibration choices. Conversely, we expect that feedback originating at the adjustment level will be relatively weak and short-lived. However, this does not mean that such feedback does not affect policy stability. For instance, ongoing criticism of policy settings (e.g., prices/charges set too high) could erode the very idea of a policy, with potentially destabilising effects. Alternatively, the combined feedback from a cumulative series of incremental policy reforms could enhance support for a policy instrument, helping to entrench it.

We retain well-established expectations about the link between feedback direction and policy stability (i.e., reinforcing feedback enhances stability, undermining feedback erodes stability). But we also posit that combinations of within- and across-level feedback might result in counterintuitive effects. The potential for such effects has been substantiated in formative studies on mixed feedback. For instance, different components within one policy instrument might generate competing supporting constituencies that reinforce and stabilise each component, but also trigger policy conflicts that prevent broader policy reform (Moore & Jordan, 2020). This results in the overarching policy being preserved not because it is effective, but because no one can agree on how to change it without upsetting the existing balance of benefits. By perpetuating outdated and even conflicting policy components, such “stability by stalemate” (Moore & Jordan, 2020, p.303) increases the risk of policy drift, and thus instability over time through eroded policy effectiveness. Conversely, a policy element that undermines itself might be a catalyst to reform (rather than remove) a broader policy, ultimately enhancing policy stability (Daugbjerg & Kay, 2020; Daugbjerg & Bazzan, 2024). Unpopular provisions generating undermining feedback within a broader, generally popular policy might also prove surprisingly durable because they cannot easily be replaced without also undercutting their popular (reinforcing) overarching instruments, creating an ambiguous situation that is neither purely reinforcing nor undermining (Béland et al., 2019). Finally, a policy may also have numerous reinforcing elements within its instrumentation and calibration, but still generate undermining feedback at the ideas level (for example, due to subjective perceptions about its costs), resulting in overall policy instability despite the presence of seemingly stable instruments.

The potential for counterintuitive effects of mixed feedback also highlights the need to consider case context. Exogenous factors such as wider political factors, institutions, and external events can mediate feedback (Béland et al., 2022; Lockwood, 2022; Patashnik & Zelizer, 2013). For example, increasingly polarised partisan identities can mute the importance of a policy’s material costs/benefits and elevate the importance of elite cues in shaping feedback (Hacker & Pierson, 2019). Focusing events like financial crises and natural disasters can similarly change how policy costs and benefits are perceived (Daugbjerg & Kay, 2020). Exogenous factors can also shift the balance of mixed feedback (Oberlander & Weaver, 2015). Altogether, it is therefore difficult to predict the effects of mixed policy feedback, requiring in-depth case-specific empirical analysis.

## Methodology

The domain of climate change mitigation, particularly carbon markets, is salient for studying mixed policy feedback for several reasons. First, undermining feedback could be prevalent. Climate policy is frequently contested, especially because it often comes in the form of classic general interest reforms: imposing short-term concentrated costs in exchange for diffuse and uncertain long-term benefits (Patterson, 2023; Patashnik, 2008). Both policy proponents and opponents often view climate mitigation as high-stakes, further increasing the chance of policy conflict. Scholars have posited a need to design climate policies that “intentionally stick” through reinforcing feedback (Jordan & Matt, 2014), such as by creating new beneficiaries (Meckling, 2019). Moreover, shifting public opinion and climate policy preferences over time, or normalisation of policy expectations after a policy is introduced, might drive policy feedback in time-varying ways. In such situations, the composition of mixed policy feedback and its effects on policy stability are especially unclear.

### Case selection and backgrounds

We examine how mixed policy feedback affects policy stability in two complementary cases of contested national carbon market regimes in Australia and Canada. The Australian case focuses on a baseline and credit scheme for industrial emissions (a type of carbon market policy) (2014–2025), and the Canadian case focuses on a national carbon pricing policy (2015–2025). We selected these policies because they allow us to focus on the contentious ‘post-enactment’ stages of policymaking (Stokes, 2020) in contemporaneous and similar national contexts. Both countries share similarities, including a Westminster and federalist political systems, significant economic dependency on fossil fuels, and similar social and economic development statuses, population sizes, and geographical characteristics. Reflecting their market liberal economic systems, climate mitigation policy debates in both countries have largely centred on markets and pricing. Nonetheless, climate policy trajectories have varied between the cases (see backgrounds below). Although the cases are similar in several ways, offering opportunities for comparison, we do not explain empirical outcomes strictly in comparative terms. Instead, we use the two cases as parallel in-depth analyses offering common and complementary insights about mixed policy feedback and its effect on policy stability. This also helps to address the need for policy feedback studies to move beyond single-country cases (Béland & Schlager, 2019).

#### Case 1: Australia’s baseline and credit scheme

**Fig. 3:**
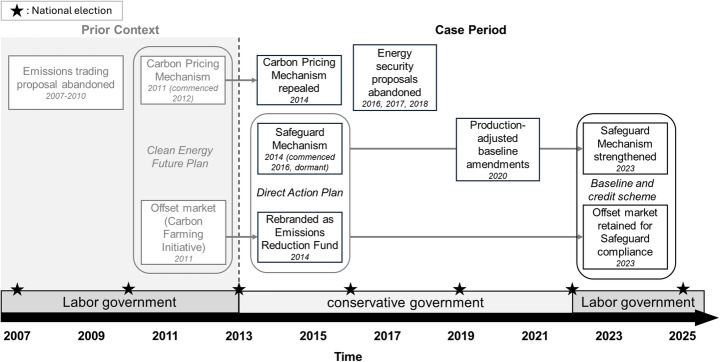
Timeline of policy developments in Australia’s baseline and credit scheme, including prior policy context

Australia’s climate policy was highly volatile following the acrimonious repeal of its carbon pricing scheme in 2014, and it remained so for almost a decade of conservative government. Nevertheless, its baseline and credit scheme, comprising a carbon offset market paired with industrial emissions baselines (the Safeguard Mechanism), endured and was later strengthened. Figure 3 shows a timeline of key policy developments (2013–2025).

Australia’s scheme was created amidst the “climate wars” (ABC, 2020), a period of intense debate around carbon pricing. Conservative-led hostility towards carbon pricing intensified after the Labor (centre-left) government successfully introduced a carbon price in 2011. Although it was actually an emissions trading scheme, critics successfully framed the carbon price as a costly, ideologically undesirable ‘carbon tax’. Other aspects of Labor’s policy were less controversial. A key component of the subsequent baseline and credit scheme, a carbon offset market, was initially introduced to complement carbon pricing and was generally well-liked. However, ‘carbon tax’ backlash was definitive. The Liberal-National Coalition (hereafter referred to as the conservatives) won a strong majority in 2013 with a central promise to scrap carbon pricing.

After repealing the carbon price, the conservatives replaced it with a rebranded and expanded offset market, the Emissions Reduction Fund (ERF). Instead of pricing pollution, this used reverse auctions to purchase carbon offsets (P1, P3, P22, P32). Bolder ideas, including several energy security proposals with plans to regulate industrial emissions intensity, were killed off by party infighting. Nonetheless, some politicians still believed voluntary offsets alone were insufficient, so they included the Safeguard Mechanism within wider ERF legislation (P6, P10, P22). This Mechanism regulated large emitters by monitoring their emissions and setting performance-based emissions limits (Power, 2018). Combining it with an offset market functionally created a baseline and credit scheme (a type of carbon market instrument). However, this system sat mostly idle for years, except for tweaks to streamline compliance (e.g., production-adjusted baselines). It was not until 2023 that a newly elected Labor government strengthened the scheme by significantly lowering its emissions limits (Gibson et al., 2024).

#### Case 2: Canada’s carbon pricing scheme

**Fig. 4:**
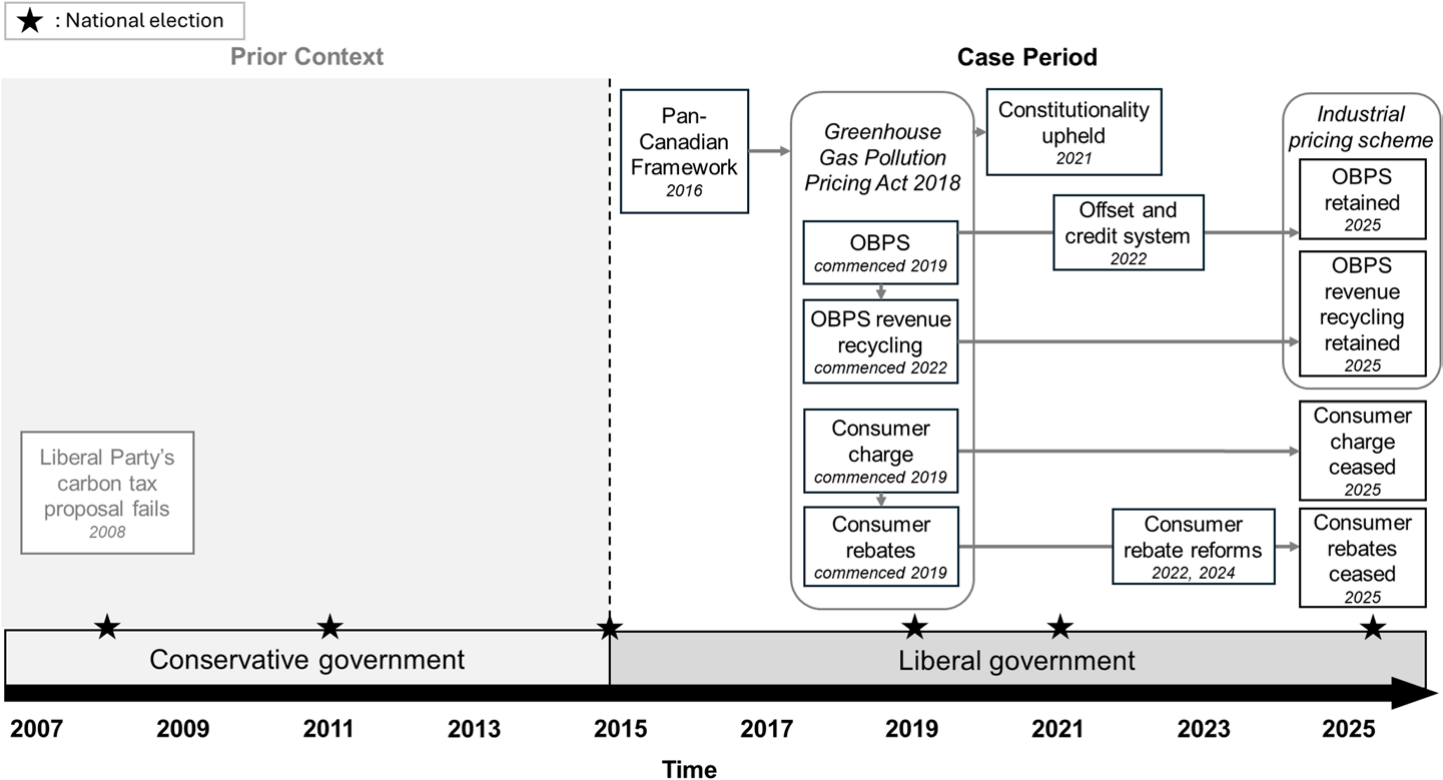
Timeline of policy developments in Canada’s carbon pricing scheme, including prior policy context

Canada’s carbon pricing scheme was built on two main pillars: a consumer charge (a charge on retail fuel like gasoline) and an Output-Based Pricing System (OBPS) that set emissions efficiency benchmarks for high-emitting facilities. This scheme survived political and legal challenges for a longer period, although the consumer charge was eventually ceased. Figure 4 shows a timeline of key policy developments (2015–2025).

Canada’s national carbon price was born in a politically and regionally divisive context. The previous Conservative government implemented no meaningful climate policy and was seen as climate change sceptical. At the 2008 election, they crushed a carbon tax proposal made by the opposing Liberal (centre-left) Party, making carbon pricing a poisoned chalice among progressives for many years (P52, P53; CBC, 2008). Nonetheless, this changed in 2015 when the Liberals were elected on a pro-carbon pricing platform (MacNeil & Paterson, 2018). The Liberals seized on a rare political alignment between federal and provincial governments, particularly in Ontario and Alberta (P36, P37, P40, P42, P44, P49), to develop national-level carbon pricing. Provincial support was vital because Canadian provinces wield strong constitutional powers and maintain distinct identities, from the conservative-leaning, oil-rich prairie provinces to the uniquely francophone Quebec. The idea of carbon pricing also had international momentum and, at first, experienced little pushback from the national Conservatives in opposition.

Canada’s carbon pricing scheme evolved through several stages rooted in this context. In 2016, the Pan-Canadian Framework on Clean Growth and Climate Change established the principles of revenue neutrality and a “benchmark and backstop” approach (ECCC, 2017): the federal government would only intervene if a province’s own policy was not ambitious enough, or if provinces chose to opt in. The 2018 Greenhouse Gas Pollution Pricing Act formalised the scheme’s two core instruments, which kicked in from 2019. Over the next few years, minor amendments and programs, such as an offset and credit system and consumer rebate reforms, were introduced to ease costs and clarify public benefits.

The Canadian scheme remained stable for almost a decade, despite prior divisiveness and waves of opposition (Bognar et al., 2020). Opposing provinces^2^ challenged its constitutionality, but the Supreme Court affirmed the national carbon price was valid in 2021. The governing Liberals won two more elections (2019 and 2021) despite the Conservatives’ anti-carbon pricing campaigns (P36, P49, P44). However, opposition persisted, especially targeting the consumer charge and weaponising yearly price increases to stir public anger (e.g., Horwood, 2024). Despite this, the carbon price survived and actually expanded: by late 2024, the consumer charge covered ten of Canada’s thirteen provinces and territories (P39, P46; ECCC, 2024).

The scheme eventually destabilised, but much later than Australia’s. In early 2025, Prime Minister Trudeau stepped down as Liberal leader. Simultaneously, the Conservative opposition was successfully targeting the unpopular consumer charge and climbing in the polls. They seemed set to win the 2025 election (Global News, 2024a). To stop it from becoming an election wedge, the new Liberal Prime Minister, Mark Carney, abolished the consumer charge in April 2025 (Canadian Press, 2025). The industrial scheme remained in effect.

### Data collection and analysis

We employ a qualitative interpretive approach. We draw on semi-structured interviews with policy experts (*n* = 68) conducted between January and April 2023, in-person (in both countries) and virtually. Participants, cited as P[X] for anonymity, are elite actors with direct experience and/or close knowledge about each respective case, including policymakers, political actors, interest groups, and academics (Table 1). We also draw on publicly available policy documents (e.g., budget records, departmental websites) and prior academic accounts. Our study was approved by the Ethics Review Board of our institution (GEO S-22878, December 16, 2022).

**Table 1:** Overview of participants and their roles

Type	Role	Participants	
		Case 1: Australia’s baseline and credit scheme	Case 2: Canada’s carbon pricing scheme
Policymakers	Political actor	P3, P15*, P18*, P19*, P21*, P32*, P38*	P42*, P46*, P49, P55*, P62*, P67*
	Central agency	P1*, P6, P8*, P16*, P17*, P26*, P27*, P28*, P29*, P33	P41, P44*, P45*, P57*, P65*
	Climate, energy, and/or environmental policy agency	P1*, P2, P4, P5, P7, P8*, P9, P10*, P11, P12*, P13*, P14, P16*, P17*, P18*, P19*, P20*, P22, P24, P25*, P26*, P27*, P28*, P30*, P31*, P34, P35*, P38*	P37*, P39, P40*,P43, P44*, P45*, P47*, P48, P51*, P52*, P54*, P56*, P57*, P58*, P59, P60, P61*, P62*, P63, P65*, P66*, P67*
Interest groups	Think tank	P12*, P13*, P15*	P42*, P46*, P50*, P52*, P53*, P55*, P56*, P58*, P64*, P65*
	Lobby group	P21*	-
Academics	-	P10*, P16*, P17*, P20*, P23, P25*, P30*, P31*	P36, P37*, P40*, P45*, P47*, P50*, P51*, P53*, P54*, P61*, P64*, P66*, P68
Industry	Firm/consultancy	P30*, P31*, P32*, P35*	P46*, P51*, P52*, P53*, P55*, P62*
Total:		*n* = 36	*n* = 32

*Denotes participants with more than one area of case expertise

We analyse data using Nvivo, coding for (1) policy elements, (2) types of policy feedback,^3^ (3) effects on policy stability, and (4) contextual/exogenous factors. In Section 4, we begin each case with a snapshot of key elements comprising each policy. We then explain the feedback generated by developments in these policy elements and the effects on overall policy stability. We thus reveal how different elements initiate different feedback within and across policy levels, producing mixed feedback affecting overall policy stability.

## Results

### Australia

Australia’s baseline and credit scheme involved various policy elements together comprising an approach to monitoring and flexibly reducing carbon emissions from large industry (Table 2). While reducing emissions was a publicly stated goal, the conservative government was initially most concerned with avoiding constraints and costs on industry. The result was a voluntary, incentive-based and flexible policy. Nonetheless, unfolding feedback and pressure from pro-climate actors eventually enabled the scheme to transition from voluntary to coercive. Mixed feedback arose in several ways: pre-existing anti-carbon pricing ideas continued to undermine policymaking; specific policy designs and adjustments triggered new reinforcing and undermining feedback, and yet other feedback was linked to changes in context. This section explains how policy developments triggered mixed feedback within and across policy levels, and how this overall impacted policy stability. Table S1 (Supplementary Materials) provides a further detailed summary of feedback types, including mechanisms, timeframes, direction, strength, and effect on policy stability.

**Table 2:** Policy elements of Australia’s baseline and credit scheme (2014–2025)

Policy level	Policy elements	
	Aims	Means
Ideas	*Goals*	*Logics*
	Avoid coercive policy Avoid imposing costs Meet emissions reduction goals (initially weak, later increased)	Voluntary action Incrementalism
Instrumentation	*Objectives*	*Instruments*
	Non-binding and voluntary instruments, later strengthened Co-benefits where possible	Safeguard Mechanism (non-binding, eventually made binding) Expanded offsets market
Adjustment	*Settings*	*Calibrations*
	Flexible emissions baselines	Several options for baseline calculations, including production-adjusted baselines

#### Ideas level feedbacks

Prior conflict, especially over the short-lived carbon price (the Carbon Pricing Mechanism), triggered persistent undermining feedback that poisoned future climate policymaking. During this time, carbon pricing supporters were unable to counter the ‘axe the tax’ criticism mobilised by opponents (Crowley, 2017; Fenna, 2023). The policy’s public benefits remained too abstract, and its direct beneficiaries, like the renewable energy industry, lacked the power to defend it. Even direct payments for affected industries and households failed to build support (P1), partly because the government “strange[ly]” (P17) refused to link compensation to the carbon price. Like many general interest reforms (Patashnik, 2008), the carbon price ultimately failed to generate enough reinforcing effects to survive political attack.

Even after the carbon ‘tax’ was scrapped, its legacy undermined further appetite for bold climate action. Once in power, most conservative elites focused not on policy content or effectiveness, but on avoiding any policy that resembled a tax (P22). They attacked new ideas as “bringing back the tax” (P13) and “carbon pricing in disguise” (P35, supported by P1, P4, P16, P17, P24, P26, P31). This became so intense that it even contributed to the 2018 toppling of a sitting Prime Minister (Murphy, 2019). The only policy idea that was not shut down was expanding and renaming Australia’s voluntary carbon offsets market into the Emissions Reduction Fund. This emphasised “carrots rather than sticks” (P31) in a clear contrast to the previous carbon tax (P1, P3, P13, P21, P22, P24, P32). Meanwhile, some politicians and bureaucrats continued to work towards raising policy ambition. They avoided hostility to carbon pricing by focusing on smaller and technical tweaks at policy levels less subject to scrutiny (P20, P24). As one senior bureaucrat stated: “…if we’re not going to solve it with just a [carbon] price…is there a sequence of steps that are small or not very big in their own right but actually that take us on a path?” (P1).

#### Instrumentation level feedbacks

The Safeguard Mechanism was key to this incremental approach, with several features aimed at quietly reinforcing it while avoiding political scrutiny. First, its creators carefully named it to distance it from carbon pricing and presented it to the Cabinet as a harmless monitoring scheme (P1, P3, P9, P16, P17, P33, P35). Although it contained regulatory ‘sticks’, the government publicly described it as “an element” (P13) of the Emissions Reduction Fund to divert attention towards voluntary offset ‘carrots’ (P1, P18). Bureaucrats carefully maintained a low-key, anti-tax framing when briefing sceptical politicians about the Mechanism (P12, P27). All of these efforts fit what Stokes (2020, p.49) calls strategic “fog of enactment”, where advocates intentionally obscure the possible impacts of a policy to avoid triggering opposition. The instrument was also harmless in practice as well as in name. While its creators always expected it to “evolve…so that the dial could be turned and tightened by subsequent governments to meet higher targets” (P3), they initially set the Safeguard Mechanism’s baselines so low that the instrument was basically dormant (P1, P35). This long grace period signalled to industry that they were getting a better deal^4^ than if the Mechanism had been immediately binding (P21). Yet, it still required industry to track and report emissions, locking in the data and industry capacity needed to later give the instrument teeth (P1). Together, these features helped the Safeguard Mechanism survive. The public and politicians who might otherwise oppose the Mechanism did not know about it or thought it was too small to matter, so they did not bother to reject it. Meanwhile, its industry targets were getting used to the minimal costs of the scheme and even coming to accept the prospect of it eventually strengthening.

At the same time, the Emissions Reduction Fund also had several reinforcing features. Early on, the government highlighted its co-benefits for farmers and indigenous groups to build broad support (P1, P10). Moreover, expanding the instrument to purchase more and new carbon offset types made carbon offset businesses more influential and invested in the policy. Later, offsets were pitched as a way to minimise industry costs once the baseline and credit scheme was strengthened (P18, P21). This turned the industry regulated by the Safeguard Mechanism into supporters of the offsets market, as they saw it as a buffer against inevitably higher costs. Thus, the offsets market and Safeguard Mechanism –the two pillars of a possible baseline and credit scheme– became increasingly intertwined with reinforcing feedback.

#### Adjustment level feedbacks

The Safeguard Mechanism was incrementally adjusted to build its own support and normalise its future transition. The top priority was following the “…edict that it should not be binding…[When] we thought it might actually bind on someone…we actively changed the policies so that it wouldn’t do that” (P35). For example, the government simplified emissions baseline calculations and introduced production-adjusted baselines, which allowed industry emissions to grow as long as output efficiency improved. These adjustments reinforced industry support by lowering (perceived and actual) compliance costs (P16, P35). However, they were also increasingly noticed by some members of the public, who critiqued the Safeguard Mechanism as a pointless “smoke screen” for Australia’s lack of climate ambition (Morton, 2020). The result was a counterintuitive feedback tug-of-war. Technical tweaks reinforced the instrument, but also undermined the broader policy idea by accelerating public calls for its reform and, more broadly, fueling concerns over the government’s lack of climate ambition.

Through regulatory amendments rather than major new ideas or instruments, the Safeguard Mechanism was finally given teeth in 2023. Many conservative politicians expressed lingering opposition, but this was muted by the ‘new’ baseline and credit scheme being an amended version of a policy they had approved then ignored for almost a decade. Big industry, carbon offset providers, and the public broadly accepted the reforms. All the previous quiet tweaks mattered in reinforcing the baseline and credit scheme before it became binding. As one bureaucrat put it, “All of the stuff that we did to reform the Safeguard Mechanism… If we hadn’t done that, we wouldn’t be where we are now” (P14).

#### Effects on policy stability

As expected, ideas-level feedback had the most serious impact on policy stability. The ghost of the carbon ‘tax’ continued to undermine climate policy efforts, generating instability that was “…detrimental to long-term [policy] investment security” (P8). Yet, behind this political struggle, some policymakers still supported the idea of carbon pricing (P3, P23, P33) and took to shaping policy that could strengthen itself at more detailed policy levels through a baseline and credit scheme that quietly took root. It survived because it emphasised voluntary ‘carrots’, and its regulatory ‘sticks’ were largely invisible and initially dormant, allowing industry acceptance and data/monitoring capacity to grow. However, this also created counterintuitive effects. Notably, its flexibility and non-binding nature helped stabilise the Safeguard Mechanism instrument, but this same weakness fueled public demands for more climate action, undermining the policy’s wider stability. A changing political context led to the scheme being strengthened without reigniting the ‘climate wars’ in 2023. Years of weak climate policies intensified public calls for climate action (itself accelerated by instrument-level feedback). The 2022 change to a new Labor government provided the policy window to make these amendments. Yet, overall, the cumulative effect of smaller reinforcing feedback at detailed policy levels, although not decisive on its own, tempered dominant anti-carbon pricing ideas that led to this moment. Labor’s decisive 2025 win and the absence of the scheme from major debate suggest that the baseline and credit scheme has further stabilised.

### Canada

Canada’s carbon pricing scheme involved multiple policy elements together comprising a nationwide approach to pricing carbon emissions from consumers and industry (Table 3). This was oriented towards balancing the government’s emissions reduction goals with differing regional needs and concerns about costs, which they knew opponents would leverage. The result was a complicated policy that tried to pre-empt criticism but also experienced a range of challenges. Mixed feedback arose in several ways: existing anti-tax and provincial autonomy ideas were used by both supporters and opponents of carbon pricing to try to create feedback. Specific policy features also triggered new reinforcing and undermining feedback. This section explains how policy developments triggered mixed feedback within and across policy levels, and how this overall impacted policy stability. Table S2 (Supplementary Materials) provides a further detailed summary of feedback types, including mechanisms, timeframes, direction, strength, and effect on policy stability.

**Table 3:** Policy elements of Canada’s carbon pricing scheme (2015–2025)

Policy level	Policy elements	
	Aims	Means
Ideas	*Goals*	*Logics*
	Emissions reduction Cost-minimisation Provincial autonomy	Carbon markets Revenue neutrality Benchmark and backstop approach to ensure national consistency
Instrumentation	*Objectives*	*Instruments*
	National benchmark and backstop carbon price	Output-adjusted carbon pricing for industry Consumer fuel charge
	Revenue recycling	Industry Assistance programs Consumer rebates
	Flexible compliance	Offset Credit System
Adjustment	*Settings*	*Calibrations*
	Pricing certainty Transparent instrument linkages	Consumer rebate payment reforms Transparent pricing schedule Contracts for difference

#### Ideas level feedbacks

Both proponents and opponents used arguments about costs to try to influence public opinion on carbon pricing. The government tried to build support by highlighting the policy’s revenue neutrality (P36, P40, P44, P47, P48). At first, this “calmed the waters unbelievably” because it stopped opponents from saying that carbon pricing was a “tax grab” (P52). However, the government’s rhetorical advantage slipped as the policy became more concrete. Opponents mounted a simple but loud campaign focused on its perceived costs to regular people, linking this to more general anti-tax ideas. They focused on the consumer charge (or conflated the charge with the wider scheme), calling it a ‘tax’ and exaggerating its costs. Although the consumer charge was technically not a tax, the public could not tell the difference. Slogans like “axe the tax” (Global News 2024b) and warnings that the policy was “job-killing” (Baxter, 2019) or would increase energy bills (Macneil, 2020) proved more influential on public opinion than actual policy costs and benefits. Roughly a third of Canadians remained opposed to the consumer charge in 2025 (Tindall et al., 2025).

Canada’s strong provincial autonomy further complicated the idea of carbon pricing, with both sides trying to use it to their advantage (P44, P63). The government knew that a simple national carbon price would trigger backlash over federal overreach (P39, P40). Instead, they allowed greater provincial autonomy through the ‘benchmark and backstop’ approach and embedded this within the 2016 Pan-Canadian Framework (P47, P48). While crucial to gaining initial provincial support and to carbon pricing being constitutionally upheld, the flexibility of this approach also unintentionally undermined the policy idea. Policymakers struggled to explain the interactions and effects of the various carbon pricing instruments operating at the national and provincial levels (P39). The resulting confusion of the patchwork design enabled opponents to claim that carbon pricing was unfair because it seemed to hit some regions harder than others (P44). This amplified fear of concentrated costs alongside more general public concerns over costs, further eroding public support.

#### Instrumentation level feedbacks

The government implemented various instruments to reinforce policy support and counter cost concerns, though with mixed and sometimes counterintuitive effects. On the one hand, the Output-Based Pricing Scheme had several features that successfully garnered support. First, it was designed as an emissions intensity scheme, allowing target industries to scale production to market conditions (including increasing their emissions) as long as they were reducing per-unit emissions. The government promoted this as maintaining industrial competitiveness compared to a straight carbon tax. Support was further reinforced from 2022 through OBPS revenue payments (direct programs to industry and payments to provinces) and the addition of an offset credit system, which further reduced compliance costs and fostered new support from carbon offset businesses (P39, P63). These efforts reinforced support for the OBPS because they kept both perceived and actual costs low for industry and widened the policy’s benefits to other businesses (P39, P43, P47, P48, P49).

On the other hand, consumer charge rebates failed to consolidate public support for carbon pricing. Despite a generous design that often returned more money than households had paid, “…people just didn’t see it [and] didn’t recognise” (P46) the rebates (P37, P47, P48, P49, P51). Even when people recognised the rebates, the underlying logic –charging a fee, only to refund it later– proved difficult to explain. The scheme’s patchwork design compounded public confusion about the source and purpose of the rebates, making it more difficult for the government to directly use rebate benefits to counter cost concerns (P36, P44, P46, P55, P58). Altogether, this fits what Campbell (2012, p.346) calls informational failure, where policy benefits are “…insufficiently visible, traceable, and salient” to generate feedback.

Finally, the consumer charge continued to expand across provinces despite opposition, suggesting an ambiguous mix of feedback. The carrot of recycled revenue incentivised its expansion, as only provinces that opted in to the national-level charge could access these funds (P46, P48). However, opting in also provided a strategic advantage to opposing provinces: it allowed them to deflect blame for costs towards the federal government (P61, P63). This reveals a counterintuitive effect where the policy instrument was reinforced, even while its underlying policy idea was undermined.

#### Adjustment level feedbacks

The government tried various policy adjustments to counter dominant cost concerns, with similarly mixed success. In 2023, they introduced contracts-for-difference to stabilise expectations about the long-term future of the OBPS. By guaranteeing a longer-term fixed price for carbon credits, regardless of who was elected, these contracts solidified support from carbon offset businesses (P37, P49, P60). This also made scrapping the OBPS financially prohibitive for future governments (P50, P51, P63). Conversely, government efforts to fix informational failures in consumer charge rebates largely failed. Despite shifting payments to be more regular and direct (from yearly tax payments to quarterly bank deposits), working with banks to clarify deposit labelling, and repeatedly rebranding the rebates to try to make their link to carbon pricing more obvious (P45, P46, P48, P51), public recognition continued to be low. In 2024, only around half of eligible people realised they even received rebates, let alone linked them to carbon pricing (Coletto, 2024). This lack of perceived benefit was a policy vulnerability. Concerns about high costs and federal interference continued to dominate, and the consumer charge became increasingly unpopular, threatening to destabilise the wider policy.

Finally, setting the carbon price itself –particularly the government’s decision to announce annual price increases well in advance– created contradicting feedback. For industry, the forward schedule provided greater cost certainty, allowing them to better plan as the government intended (P37, P49). However, the forward schedule also provided an opportunity for opponents to mobilise by using annual price increases to stir public anger. Consequently, a single policy adjustment simultaneously reinforced industry support and accentuated widespread cost concerns.

#### Effects on policy stability

The carbon pricing scheme remained largely stable despite policy conflict. As expected, the most serious threats to its stability came from the ideas level, with ongoing criticism over perceived costs and impacts on provincial autonomy undermining support. However, the government anticipated opposition and sought to counter it at all policy levels, drawing on the same ideas as opponents as well as designing, implementing and adjusting policies to try to build support. Yet, not all these efforts were successful. The industrial component (OBPS) mostly reinforced and thus stabilised itself, while the consumer charge component eventually undermined and destabilised itself despite reinforcing interventions. Overall, the carbon pricing scheme initially weathered opposition but experienced an undercurrent of sometimes conflicting, mixed feedback that tempered policy stability. This included unintended and sometimes counterintuitive effects across policy levels. In particular, reinforcing dynamics at more detailed levels (the consumer charge expansion; policy certainty created by the advance pricing schedule) could simultaneously allow provinces to undermine wider policy ideas. When combined with the context of a leadership crisis and abrupt economic and sovereignty threats from the US late in 2024, the policy’s hidden vulnerabilities eventually led to the consumer charge component being retrenched.

## Discussion

**Fig. 5:**
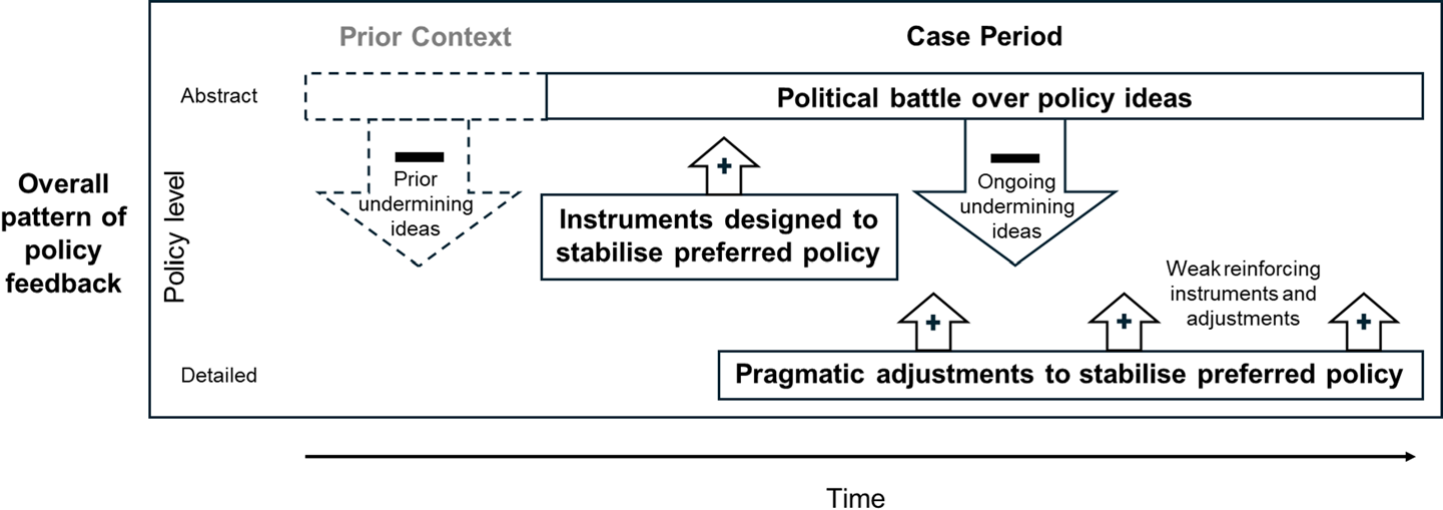
Shared overall pattern of mixed policy feedback in the two cases

Both cases showed broadly similar patterns of mixed feedback despite differing policy stability outcomes (Fig. 5). Both were preconditioned by prior policy contestation, generating ongoing interpretive feedback (linked to perceived costs and broader policy meanings) that undermined subsequent policy designs and stringency. As expected, this ideas-level feedback had the strongest and most persistent impact on overall policy stability. To counter this, proponents in both cases tried to design, frame and refine policies that built supportive constituencies (publics, industry) and maintained economic competitiveness. Yet, the weaker and often shorter-lived reinforcing feedback triggered by these efforts only partly countered dominant undermining ideas. In effect, we saw competition between both policy levels and types of feedback mechanisms, with undermining interpretive feedback at the ideas level being countered by reinforcing (largely resource) feedback at more detailed levels.

**Fig. 6:**
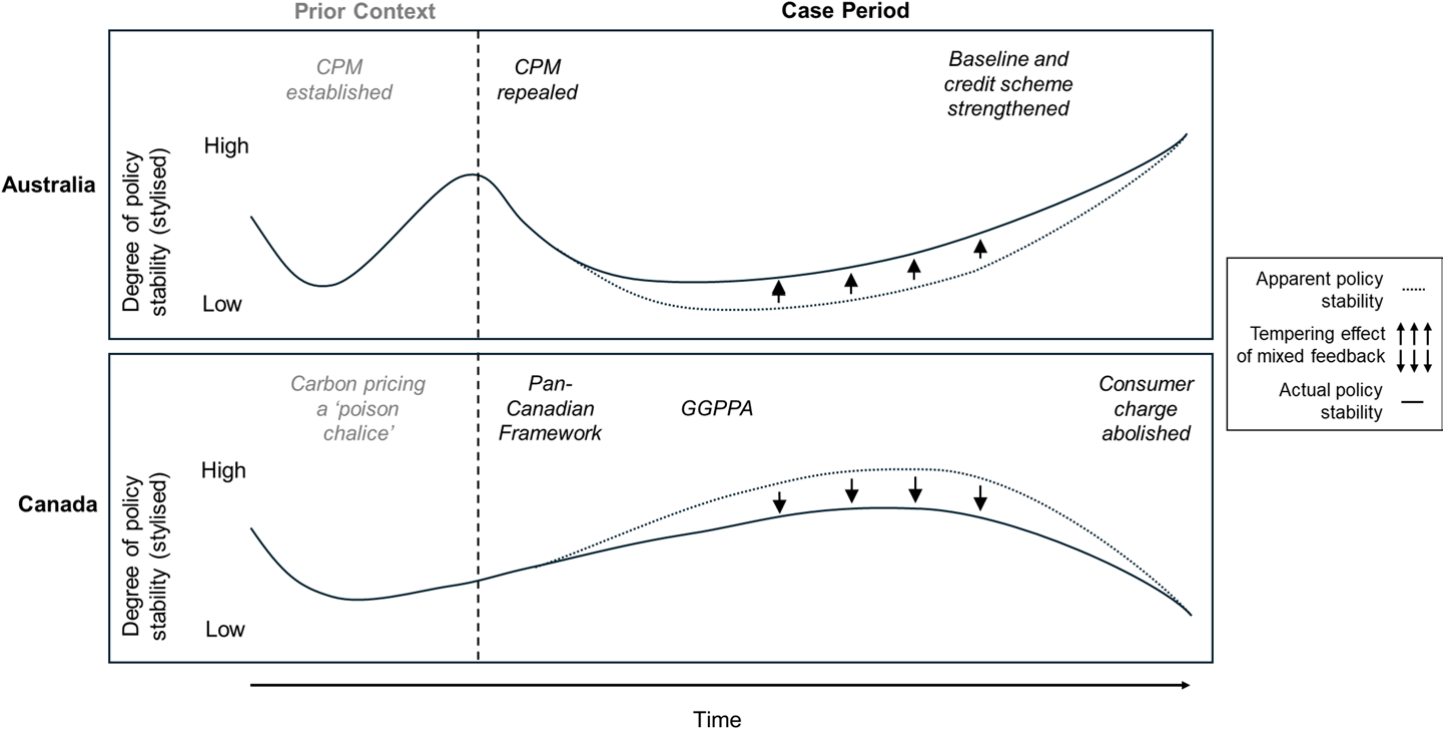
The tempering effect of mixed policy feedback on policy stability in both cases

Overall, mixed policy feedback tempered apparent policy stability in both cases (Fig. 6), rendering it more tenuous than it seemed. Outwardly visible feedback (undermining in Australia, reinforcing in Canada) was counteracted by less obvious but not less consequential feedback in opposing directions. In Australia, continued ideological rejection of policy costs and coercion led proponents to craft and maintain an alternative to carbon pricing that was weak and covert enough to avoid, and even counter, undermining feedback. Incremental efforts initially seemed fruitless but gradually helped to entrench the Safeguard Mechanism, enabling its eventual strengthening. In Canada, culturally rooted ideas around cost aversion and provincial autonomy were used to both defend and attack carbon pricing, driving competing interpretive feedback. Policymakers also tried to reinforce the policy at more detailed levels, and the policy seemingly remained stable as a result. Nonetheless, persistently undermining ideas quietly eroded the policy to the point that the consumer charge was vulnerable to removal by its own supporters. Overall, this suggests mixed policy feedback can have a hidden tempering effect: creating unexpected sources of stability where this is lacking, or unforeseen fragility in what otherwise seems to be a stable policy.

Our findings suggest a complex relationship between mixed feedback and policy stability. While dominant undermining ideas were partly countered by weaker, lower-level reinforcing feedback in both cases, the resulting variation in policy stability outcomes was not just a ‘net effect’. The tempering effect suggests there is ‘more going on under the hood’ than apparent policy stability indicates. Policy stability depends on how specific feedbacks combine and interact within and across policy levels. Importantly, in both cases, mixed feedback arose intentionally but also unintentionally, with the latter disrupting efforts to simply counter undermining feedback with reinforcing feedback. Moreover, exogenous factors in the wider context (e.g., elections) influenced policy windows through which policies primed by feedback became more or less stable. Consistent with prior work on mixed feedback (e.g., Geels & Ayoub, 2025; Béland et al., 2019), the resulting policy stability outcomes were linked to (but not fully determined by) the specific combination of feedback in each case.

These findings also extend research on the counterintuitive effects of mixed policy feedback. First, we demonstrate a new way in which a single policy element can have counterintuitive effects on policy stability across policy levels. In Australia, the Safeguard Mechanism’s flexible and non-binding nature stabilised the instrument by keeping industry on board, yet undermined broader policy stability by fueling public demands for greater climate ambition. Likewise, Canada’s pre-announced carbon price schedule provided policy certainty that helped entrench industry support for carbon pricing, but also gave opponents an opportunity to undermine policy ideas. Prior studies of mixed feedback show that undermining policy instruments can promote policy stability if they lead to adaptation in policy ideas (Daugbjerg & Kay, 2020; Daugbjerg & Bazzan, 2024). We identify an inverse effect: reinforcing instruments can undermine a policy idea by catalysing calls for reform. This could arise when reinforcing instruments become overly rigid, preventing broader policy adaptation (Daugbjerg & Kay, 2020). Our case examples reveal another causal pathway, where instruments that reinforce themselves with one constituency can simultaneously provoke ideas-level opposition from another. This underscores that counterintuitive mixed feedback can stem from the conflicting motivations between different policy actors. Analysing mixed feedback arising at different policy levels helps to reveal such dynamics.

The somewhat surprising expansion of Canada’s consumer charge across provinces and territories reveals another counterintuitive effect, with implications for how mixed feedback is analysed. Based on policy features alone, it would be easy to conclude that incentives to receive recycled revenue (a classic reinforcing mechanism) drove this expansion, but interviewees raised that some provinces also likely opted in to divert blame towards the federal government. Thus, ambiguity in how mixed feedback affects policy stability can arise when the key actors involved hold dual or even conflicting motivations at different policy levels. Altogether, this highlights the role of strategic actors in feedback analysis. Mixed policy feedback is not merely a mechanistic process stemming from policy features, but also a political resource used by different actors in struggles over policy meaning and direction (Ampe et al., 2021).

Finally, our findings also underscore the importance of exogenous factors (e.g., electoral cycles, economic conditions, political cultures, and wider political developments) in shaping mixed policy feedback and its effects (Beland et al., 2022). Entrenched ‘anti-tax’ sentiments were important in both cases, stimulating persistent undermining feedback. Elections also played key roles, with climate policy varying in prominence. Moreover, feedback also primed policies for change in combination with policy windows at important moments in both cases (i.e., change of government in Australia; impending election with external pressure in Canada). Hence, analysing mixed policy feedback requires considering not only endogenous feedback but also its interaction with wider contexts and contingent developments.

## Conclusion

Our approach enables a systematic, comparative study of mixed policy feedback and its effects on policy stability by disaggregating policy elements and feedbacks within and between them. In two cases of contested climate policy, we observed that undermining ideas-level feedback had the largest impact on policy stability, while several weaker reinforcing feedbacks from specific instruments and policy settings only partly compensated. Reinforcing and undermining feedback operated simultaneously, creating complex and sometimes unexpected effects on policy stability. This aligns with emerging work on mixed policy feedback that similarly identifies the potential for counterintuitive effects (e.g., Moore & Jordan, 2020; Daugbjerg & Bazzan, 2024). Interestingly, this also suggests competition between types of feedback mechanisms, where undermining interpretive feedback is countered by reinforcing resource feedback – a competition between ideas and interests. In our cases, ideas broadly retained the upper hand among publics, but interests seemed to carry more weight with industry. This could indicate that both interests and interpretations are important among publics, whereas interests are most important among industry. In turn, future policy feedback design efforts may need an asymmetric focus that considers which mechanism types and policy levels are most effective for different policy clienteles.

We observed that mixed policy feedback tempers the degree of policy stability/instability, rendering it more indeterminate than it might otherwise appear. Actual policy stability was lower than it outwardly seemed in Canada and higher than it outwardly seemed in Australia. Yet, this was not just a matter of ‘net effects’ because complex interactions, varying effects of feedback on different actors, and possibly also more fundamental incommensurability between resource and interpretive feedbacks, mean that the effects of mixed policy feedback need to be assessed in a case-specific way. Case-specific exogenous factors are also important alongside and in connection with various feedbacks and may change their importance and operation. While existing scholarship (e.g, Béland et al., 2019) suggests that reinforcing and undermining feedback can offset each other, we argue that the effect of mixed feedback on policy stability may be deeply ambiguous. Apparent stability or instability may not be a clear indicator of underlying dynamics yet to fully manifest. In other words, an appearance of stability can be deceptive. This nuances the foundational “lock-in” (Pierson, 1993, p.606) metaphor in feedback scholarship by suggesting that even apparently locked-in policy may be hollowed out beneath the surface in ways that only become clear later (such as in combination with an exogenous pressure that reveals vulnerabilities previously overlooked). Even seemingly stable policies can remain active sites of “combat” (Stokes, 2020, p.5), where opponents keep looking for the right moment to short-circuit feedback. On the other hand, apparent policy instability may mask unfolding policy entrenchment, meaning that there is still hope for policy proponents in unstable policy situations. Policy instability spurred by conflicting ideas at the highest level is evidently challenging, but it also might be an opportunity in disguise, with loud political theatre obscuring quieter and incremental efforts to eventually stabilise the overall policy.

The rich mixed feedback we observed defies standard accounts of policy feedback as either reinforcing or undermining. This may, in fact, be the norm in climate policy, where structural problem features (e.g. concentrated costs versus diffuse benefits, loss aversion, and faster mobilisation against new policy by incumbents versus slower formation of new constituencies) mean that efforts to generate reinforcing feedback around new policy are harder than generating undermining feedback. This might also mean that instead of mixed feedback being the exception, wholly reinforcing or undermining feedback is the exception to be problematised.

This recognition of mixed feedback challenges optimistic, reinforcing transition narratives, because deliberate efforts to stabilise ambitious policies are likely to continue to encounter undermining feedback into the future. While scholars often hope that climate policy designs will inherently generate reinforcing feedback (Roberts et al., 2018), our cases show that anticipating and countering undermining feedback is difficult, especially when conflicting ideas overwhelm design intent. Nevertheless, as seen in both our cases, difficult policy changes can endure when policymakers strategically cultivate feedback, even amid ongoing policy conflict.

Overall, this highlights a key need to further examine how different actors go about trying to generate feedback, and counter each other’s moves to do so. In other words, the actor politics of generating *and anticipating* feedback. This might be as much about trying to prevent an opponent’s feedbacks from consolidating as generating one’s own intended feedbacks. For example, climate policy proponents might try to prevent undermining interpretive feedback from consolidating around an idea of costs being perceived as a tax, but opponents might try to prevent reinforcing resource feedback from consolidating support. This points to the hidden agency and political struggles involved in shaping feedback, including in relation to wider political developments. The “power struggles of actors” (Ampe et al., 2021, p.580) in shaping feedback are likely to be especially crucial in conflicted policy domains like climate mitigation, where intense undermining feedback frequently threatens policy stability. This compels scholars to look more closely ‘under the hood’ of policy feedback, at the drama behind the scenes as well as on the political stage.

Finally, we focused on carbon pricing, which leaves open a need to examine other instrument types (in climate policy, and beyond) that may reveal different patterns of mixed policy feedback. For example, framework policies (e.g. targets, strategies) or less coercive policies (e.g., investment and incentive-oriented) may be less prone to undermining feedback. Conversely, coercive but more targeted policies would still generate undermining feedback, but more targeted policy losses might make effects on policy stability less decisive, or increase the relative role of exogenous factors (e.g., public support, relative political salience of opposition). Thus, mixed policy feedback offers an exciting future agenda for studying the politics of policy feedback.

## Supplementary Information

Below is the link to the electronic supplementary material.

Supplementary Material 1

## Data Availability

The datasets generated and analysed during the current study are not publicly available due to the risk that research participants could be inadvertently identifiable.

## Footnotes

1 We use the terms “reinforcing” and “undermining” for our analysis because the common alternatives, “positive” and “negative” policy feedback, are ambiguous (Beland et al., 2022).

2 Notably, Ontario and Alberta, which had by then re-elected conservative governments.

3 We coded feedback by mechanisms, timeframes, direction and strength, consistent with Béland et al.’s (2022) recent synthesis.

4 Essentially, a mirror of the typically undermining effect that concentrated losses can trigger.
