# Supplementary material for: Tenuous (in)stability? Mixed policy feedback and its effects on climate policy in Australia and Canada
Source: Policy Sci. 2026 Apr 29;59(2):325–46. doi: 10.1007/s11077-026-09609-9 (PMC13234078; doi:10.1007/s11077-026-09609-9)
Supplement: Supplementary file 1 — Supplementary Material 1 [file 11077_2026_9609_MOESM1_ESM.docx]

**Supplementary Materials**

**Table S1:** Summary of mixed policy feedback in Australia’s baseline and credit scheme.

| **Policy level** | **Policy development initiating feedback** | **Feedback arising**  **(🡪across-level effect)** | **Feedback mechanism*** | **Timeframe** | **Direction** | **Strength** | **Effect on stability** |
| --- | --- | --- | --- | --- | --- | --- | --- |
| IDEAS | Anti-carbon tax framing forms in response to prior climate policy action, also linked to wider anti-tax sentiment | Within-level | Interpretive (sociopolitical: mass and party opposition) | Began prior to case; persistent | **-** | Strong | Idea of carbon pricing continuously undermined (destabilising) |
|  |  | Across-level (🡪instrumentation) |  |  |  |  |  |
|  |  | Across-level (🡪adjustment) |  |  |  |  |  |
| INSTRUMENTATION | Safeguard Mechanism introduced and framed as voluntary/minor | Across-level (🡪ideas) | Interpretive (sociopolitical: mass and party support) | 2014-2023 | + | Weak | Safeguard Mechanism approved by Cabinet and shielded from scrutiny (stabilising) |
|  |  | Within-level | Interpretive (informational: elite attention overload) |  |  |  |  |
|  |  | Across-level (🡪adjustment) |  |  |  |  |  |
|  | Safeguard Mechanism implemented as weak/dormant instead of coercive | Across-level (🡪adjustment) | Resource (economic: increasing returns) | 2014-2023 | + | Weak | Increased acceptance, normalisation, and eventually support among industry targets (stabilising) |
|  |  |  | Resource and interpretive (sociopolitical: concentrated benefits) |  |  |  |  |
|  | Expanded offset market designed and framed with co-benefits for farmers and indigenous groups | Within-level | Interpretive (sociopolitical: mass support) | 2014-2022 | + | Weak | Co-benefits to culturally important/deserving clientele reinforce overall policy support (stabilising) |
|  | Offset market expansion adds newly accredited offset sources | Within-level | Resource (sociopolitical: concentrated benefits) | 2015- | + | Weak | Supportive constituency of offset providers expanded (stabilising) |
|  | Offset market linked to Safeguard Mechanism as a cost-minimisation solution for industry targets | Within-level | Resource and interpretive (sociopolitical: concentrated benefits) | 2019- | + | Weak | Support among industry targets reinforced (stabilising) |
| ADJUSTMENT | Safeguard Mechanism baseline amendments | Across-level (🡪instrumentation) | Resource and interpretive (sociopolitical: concentrated benefits) | 2019- | + | Weak | Support among industry targets reinforced (stabilising) |
|  |  | Across-level (🡪ideas) | Interpretive (sociopolitical: mass opposition) |  | - | Weak | Broader (exogenous) demands for greater climate action accelerated (destabilising) |

* Mechanism types draw on Pierson (1993)’s formative distinction between resource and interpretive feedback and Béland et al.’s (2022, pp.41-65) recent synthesis.

**Table S2:** Summary of mixed policy feedback in Canada’s carbon pricing scheme.

| **Policy level** | **Policy development initiating feedback** | **Feedback arising**  **(🡪across-level effect)** | **Feedback mechanism*** | **Timeframe** | **Direction** | **Strength** | **Effect on stability** |
| --- | --- | --- | --- | --- | --- | --- | --- |
| IDEAS | Revenue neutrality goal | Within-level | Interpretive (sociopolitical: mass support) | 2015- | **+** | Weak | Reduces potency of criticism based on anti-tax sentiments (stabilising) |
|  |  | Across-level (🡪instrumentation) |  |  |  |  |  |
|  | Anti-carbon tax framing forms in response to policy action, also linked to wider anti-tax sentiment | Within-level | Interpretive (sociopolitical: mass and party opposition) | Began prior to case; persistent | **-** | Strong | Idea of carbon pricing continuously undermined (destabilising) |
|  |  | Across-level (🡪instrumentation) |  |  |  |  |  |
|  | Benchmark & backstop approach emphasises provincial autonomy | Within-level | Interpretive (sociopolitical: mass and party support) | 2015- | **+** | Weak | Provincial sovereignty concerns reduced (stabilising) |
|  |  | Across-level (🡪instrumentation) |  |  |  |  |  |
|  | Benchmark & backstop approach increases policy complexity (implementation and communication issues) | Across-level (🡪instrumentation) | Resource and interpretive (state capacity: means-end mismatch) | 2018-2025 | **-** | Weak | Perceptions of loss and unfairness grow (destabilising) |
|  |  | Within-level | Interpretive (sociopolitical: concentrated losses) |  |  | Weak |  |
| INSTRUMENTATION | Industry scheme (OBPS) introduced and framed as intensity-based price instead of carbon tax | Across-level (🡪ideas) | Resource and interpretive (sociopolitical: concentrated benefits) | 2018- | **+** | Medium | Target industry support reinforced (stabilising) |
|  |  | Within-level |  |  |  |  |  |
|  | OBPS revenue recycling programs | Within-level | Resource and interpretive (sociopolitical: concentrated benefits) | 2019- | **+** | Strong | Target industry and provincial support reinforced (stabilising) |
|  | OBPS offset crediting scheme reduces industry compliance costs and creates new offset provider constituency | Across-level (🡪ideas) | Resource and interpretive (sociopolitical: concentrated benefits) | 2022- | **+** | Medium | Target industry and offset provider constituency support reinforced (stabilising) |
|  |  | Within-level |  |  |  |  |  |
|  | Consumer charge rebate scheme | Within-level | Resource and interpretive (sociopolitical: mass support) | 2018-2025 | **+** | Weak (non-existent) | Public support not reinforced as intended (destabilising) |
|  |  |  | Resource and interpretive (state capacity: means-ends mismatch) |  | **-** | Weak |  |
|  | Provinces increasingly opt into consumer charge backstop | Within-level | Resource (sociopolitical: concentrated benefit) | 2018-2025 | + | Weak | Province’s acceptance reinforced by recycled revenue (stabilising) |
|  |  | Across-level (🡪ideas) | Interpretive (sociopolitical: mass and party opposition) |  | **-** | Weak | Public support undermined by provinces’ blame-shifting (destabilising) |
| ADJUSTMENT | Contracts for Difference scheme for offset units under the OBPS | Across-level (🡪instrumentation) | Resource and interpretive (sociopolitical: concentrated benefits) | 2023- | **+** | Strong | Supportive constituencies reinforced by future policy certainty (stabilising) |
|  |  |  | Resource (economic: increasing returns) |  |  |  | Policy lock-in via future government financial liability (stabilising) |
|  | Measures to rectify visibility and public understanding of consumer rebates | Across-level (🡪instrumentation) | Interpretive (sociopolitical: mass support) | 2019-2025 | **+** | Weak (non-existent) | Public support not reinforced (destabilising) |
|  | Forward carbon price schedule released well in advance of rises | Across-level (🡪instrumentation) | Resource (economic: increasing returns) | 2019- | **+** | Medium | Policy adapted to, normalised among  OBPS target industry (stabilising) |
|  |  | Across-level (🡪ideas) | Interpretive (perceptions of widespread loss) |  | **-** | Weak | Mobilisation of opponents undermines public support (destabilising) |
|  |  |  | Interpretive (informational: punctuated attention) |  |  |  |  |

* Mechanism types draw on Pierson (1993)’s formative distinction between resource and interpretive feedback and Béland et al.’s (2022, pp.41-65) recent synthesis.
